# Fertility outcomes in women after controlled ovarian stimulation with gonadotropin releasing hormone agonist long protocol: fresh *versus* frozen embryo transfer

**DOI:** 10.1186/s12884-021-03698-5

**Published:** 2021-03-12

**Authors:** Xiaoyan Ding, Jingwei Yang, Lan Li, Na Yang, Ling Lan, Guoning Huang, Hong Ye

**Affiliations:** Chongqing Key Laboratory of Human Embryo Engineering, Chongqing Clinical Research Center for Reproductive Medicine, Reproductive and Genetics Institute, Chongqing Health Center for Women and Children, 400013 Chongqing, China

**Keywords:** Fresh embryo transfer, Frozen embryo transfer, Live birth rate, Gonadotropin releasing hormone agonist long protocol, In vitro fertilization

## Abstract

**Background:**

Along with progress in embryo cryopreservation, especially the vitrification, freeze all strategy has become more acceptable than ever. Some studies have found comparable or higher live birth rate with frozen embryo transfer (FET) than with fresh embryo transfer(ET)in gonadotropin releasing hormone antagonist (GnRH-ant) protocol. However from our literature research, there have been no reports about live birth rate comparison between fresh ET and FET with gonadotropin releasing hormone agonist (GnRH-a) long protocol. The aim of this study is to retrospectively investigate whether patients benefit from freeze all strategy in GnRH-a protocol using real-world data.

**Methods:**

This is a retrospective cohort study, in which women undergoing fresh ET or FET with GnRH-a long protocol at Chongqing Reproductive and Genetics Institute from January 2016 to December 2018 were evaluated. The primary outcome was live birth rate. The secondary outcomes were implantation rate, clinical pregnancy rate, pregnancy loss and ectopic pregnancy rate.

**Results:**

A total of 7,814 patients met inclusion criteria, implementing 5,216 fresh ET cycles and 2,598 FET cycles, respectively. The demographic characteristics of the patients were significantly different between fresh ET and FET groups, except BMI. After controlling for a broad range of potential confounders including age, infertility duration, BMI, AMH, number of oocytes retrieved and of available embryos, multivariate logistic regression analysis demonstrated that there was no significant difference in clinical pregnancy rate, ectopic pregnancy rate and pregnancy loss rate between two groups (all *P* > 0.05). However, the implantation rate and live birth rate in fresh ET group were significantly higher than FET group (*P* < 0.001 and *P* = 0.012, respectively).

**Conclusions:**

Under GnRH-a long protocol, compared to FET, fresh ET was associated with higher implantation rate and live birth rate in infertile patients that underwent in *vitro* fertilization (IVF). The freeze all strategy should be individualized and made with caution especially with GnRH-a long protocol.

## Background

Gonadotropin-releasing hormone agonist (GnRH-a) has been used since the early 1980 s and continuously plays an important role in controlled ovarian stimulation (COS) [1]. Currently, GnRH antagonist (GnRH-ant) protocol has gained popularity and is widely used due to its shorter treatment time, fewer injections and lower ovarian hyperstimulation syndrome (OHSS) rate than GnRH-a protocol [2]. And similar perinatal outcomes were found after the GnRH antagonist versus GnRH agonist protocols for IVF [3]. However, the standard GnRH-a long protocol is still one of the key down regulation protocols in China, due to its steady and higher clinical pregnancy rate in fresh embryo transfer (ET) [4].

The fresh ET is preferred in most IVF centers when patients have available embryos, lower OHSS risk, and no other negative factors. However, increasing evidence indicates that the supra physiologic condition caused by COS may influence the endometrial and uterine environments and lead to adverse outcomes of pregnancy [5]. Therefore, along with the implementation of vitrification and subsequent improvement of clinical outcomes, frozen embryo transfer (FET) become a very effective approach avoiding the above problem in assisted reproductive technology (ART) treatment [6]. Since the development and utilization of vitrification technology, FET has been suggested to be more optimal in efficacy and safety. It is not only used in hyper-responders to reduce OHSS rate or in pre-implantation genetic testing (PGT) patients, but has also proved to improve the reproductive outcomes of IVF treatment [7].

Several studies suggested a significantly higher live birth rate (LBR) and better perinatal outcomes in FET cycles compared with fresh ET cycles [8–10]. A multicenter randomized controlled trial (RCT) study has reported that among infertile women with the polycystic ovary syndrome, FET was associated with a higher live birth rate after first transfer than was fresh ET [10]. Some studies even supported the hypothesis of so called freeze-all strategy in IVF, in which all embryos will be frozen and no fresh transfer will be conducted, to optimize success rates [11, 12]. However, several other studies demonstrated no significant differences in obstetrical and neonatal complications and live birth rate between transfer of fresh or frozen embryos in women without polycystic ovary syndrome [13, 14]. Specially, a new systematic review and meta-analysis indicated that there were no differences in LBR by the use of FET in preference to fresh ET in the overall (non-PGT) population undergoing IVF [15].

However, the COS protocol in all the studies mentioned above or in studies included in the meta-analysis was GnRH-ant protocol. There is no report regarding the evaluation of differences between fresh ET and FET with GnRH-a protocol. The aim of our study is to evaluate the pregnancy outcomes of cryopreservation of all embryos and subsequent FET compared with fresh embryo transfer using GnRH-a long protocol.

## Materials and Methods

### Patients

This is a retrospective cohort study, in which the infertile women undergoing fresh ET or FET at Chongqing Reproductive and Genetics Institute from January 2016 to December 2018 were evaluated. All patients underwent the first IVF cycle with GnRH-a long protocol, and then underwent the transfer with two day 3 embryos either in the fresh ET or in the subsequent first FET cycles. Only freeze-all cycles because of high risk of moderate or severe OHSS [16] were included in FET group. Exclusion criteria were (1) patients’ age > 34 years old; (2) patients with the thickness of endometrium on the day of embryo transfer < 0.7 cm; (3) patients with available embryos < 2; (4) patients with blastocyst transfer or PGT cycles; and (5) chromosome abnormality and uterine malformation. All procedures of this study were approved by the Institutional Review Board of Chongqing Health Center for Women and Children.The requirement for patient informed consent was waived by the Institutional Review Board because the retrospective cohort study involved existing data and records at the time of investigation, and did not retain personal identifiers in the collected information. The study patients were divided into two groups according to fresh ET cycles or FET cycles.

### GnRH-a long protocol

From mid-luteal phase in previous menses, GnRH-a (Triptorelin 0.1 mg/d or 0.05 mg/d, Decapeptyl Ferring, Germany) was used for pituitary down regulation. After 14–18 days of GnRH-a administration, if the level of estrogen < 50 pg/ml, luteinizing hormone < 5 mIU/ml and progesterone < 1 ng/ml, a daily dose of 75–300 IU recombinant follicle stimulating hormone (rFSH), depending on the patients’ age, anti-müllerian hormone (AMH) level and antral follicle counts (AFC), was administrated. The dose of GnRH-a was remained as 0.05 mg/d, or was started at 0.1 mg/d and adjusted down to 0.05 mg/d on the Gn initiative day and continued until ovulation induction. When leading follicles reached 18 mm in diameter, an injection of 250 µg of recombinant human chorionic gonadotropin (rhCG) (Merck Serono, Italy) was given, and oocyte retrieval was performed 36 h later.

The combination of vaginal progesterone (Utrogestan 200 mg every 8 h, Besins Healthcare, Spain or Crinone 90 mg/d, Merck Serono, UK) and oral progesterone (Duphaston 10 mg twice a day, Abbott Biologicals, Netherlands) were provided for luteal phase support from the day of oocyte retrieval. In the fresh ET group, on the third day after oocyte retrieval, i.e., day 3 of the embryo culture, two good or excellent quality embryos were transferred. In the FET group, embryos in case of a freeze-all policy were vitrified on day 2 or day 3 by vitrification system. For FET transfers, artificial cycle with or without GnRH down-regulation or natural cycle were used for endometrium preparation. Luteal-phase support was started three days before FET and performed by combination of vaginal and oral progesterone. If pregnancy achieved, luteal phase support continued until 12 weeks of gestation in both groups.

### Outcomes and definitions

The primary outcome was live birth rate. The secondary outcomes were implantation rate, clinical pregnancy rate, pregnancy loss and ectopic pregnancy rate. Live birth was defined as delivery of any neonate after 28 weeks of gestation [17]. Implantation was defined as the detection of an intrauterine gestational sac using ultrasonography. Clinical pregnancy was defined as a viable pregnancy with a fetal heart activity under ultrasonography. Ectopic pregnancy was defined as the detection of a gestational sac outside the uterus. Pregnancy loss was defined as the spontaneous loss of the embryo or fetus before 28 weeks of gestation [17].

### Statistical analysis

Data analyses were carried out using the Statistical Package for the Social Science software (version 20, SPSS Inc, Chicago. IL, USA). Continuous variables were expressed as the mean ± SD. The one-sample K-S test was used to check for normality (age, BMI, infertile years, AMH, basal FSH, ovarian stimulation days, gonadotropin dose and estradiol level on hCG trigger day). The Wilcoxon rank sum test was used to assess between-group differences in continuous variables with abnormal distributions. Categorical variables were represented as the number of cases (n) and the percentage (%). Categorical variables (primary infertility, primary cause of infertility, insemination method) were analyzed by Chi-square test and Fisher’s exact test. Multivariate logistic regression analysis was used to adjust for the baseline characteristics (age, infertility duration, BMI, AMH, No. of oocytes retrieved and No. of available embryos) between the two groups to assess the effect of FET on clinical outcomes (implantation, clinical Pregnancy, ectopic pregnancy, pregnancy loss and live birth). *P* < 0.05 was considered statistically significant.

## Results

**Fig. 1:**
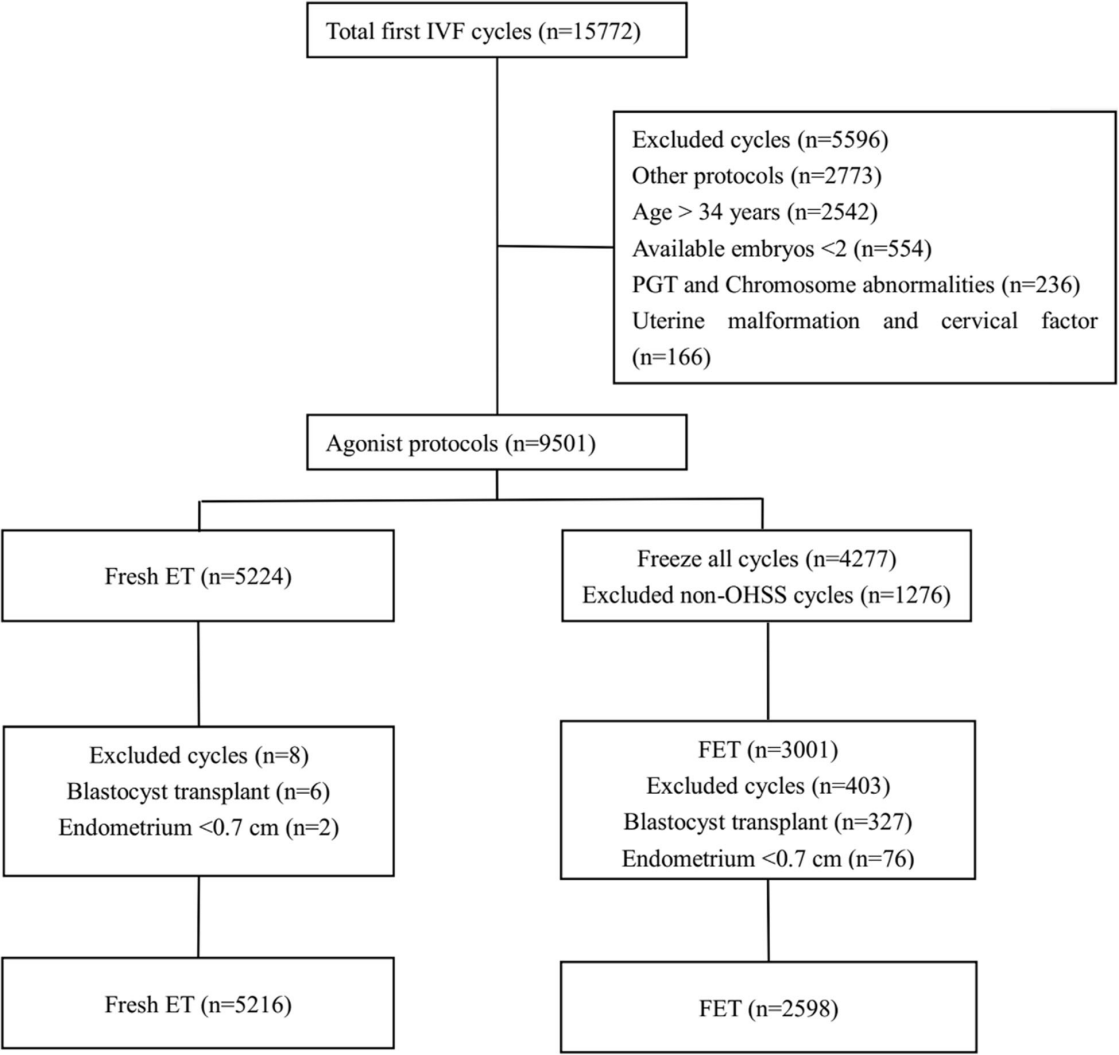
Flow chart of patient’s allocation

15,772 women were screened and assessed for eligibility. Finally, a total of 7,814 patients who underwent the first IVF cycle fulfilled study eligible criteria, including 5,216 fresh ET and 2,598 FET cycles (Fig. 1). The demographic characteristics were presented in Table 1. The age of patients was significantly older in fresh ET group than that in FET group (29.55 ± 2.86 vs. 28.96 ± 2.99 years, *P* < 0.001). The infertility duration of fresh ET group was significantly longer compared to the FET group (4.64 ± 3.07 vs. 4.43 ± 2.88 years, *P* = 0.021). There were significantly less previously nulliparous women in the fresh ET group (48.4 % vs. 51.0 %, *P* = 0.044). The basal FSH level of fresh ET group was significantly higher than that of FET group (5.64 ± 0.03 versus 5.04 ± 0.03, *P* < 0.001), while AMH level was significantly lower in fresh ET group (3.26 ± 2.54 vs. 5.58 ± 3.64, *P* < 0.001). There was no significant difference with regard to BMI between fresh ET and FET groups. Primary causes of infertility were significantly different between two groups, which was mainly due to the ratio of ovulatory obstacle (6.5 % vs. 13.4 %, *P* < 0.001).

**Table 1:** Baseline characteristics in fresh ET and FET groups

Characteristics	Fresh ET (*n* = 5216)	FET (*n* = 2598)	P
Age at egg retrieval (year)	29.55 ± 2.86	28.96 ± 2.99	< 0.001
BMI (kg/m^2^)	21.80 ± 2.74	21.74 ± 2.91	0.083
Infertility duration (year)	4.64 ± 3.07	4.43 ± 2.88	0.021
AMH (ng/ml)	3.26 ± 2.54	5.58 ± 3.64	< 0.001
Basal FSH (mIU/ml)	5.64 ± 0.03	5.04 ± 0.03	< 0.001
Primary infertility, n (%)	2522 (48.4)	1326 (51.0)	0.044
Primary cause of infertility			< 0.001
Pelvic and tubal factor, n (%)	4236 (70.2)	2040 (61.5)	
Male, n (%)	645 (12.4)	401 (15.4)	
Unexplained, n (%)	234 (4.5)	125 (4.8)	
Ovulatory obstacle, n (%)	37 (6.5)	14 (13.4)	
Endometriosis, n (%)	60 (6.4)	18 (4.8)	

Note: Presented as n (%) for categoric variables and mean SD for continuous variables. *BMI *body mass index; *AMH* Anti-Mullerian Hormone; *FSH* Follicle-Stimulating Hormone; *FET* frozen embryo transfer; *fresh ET* fresh embryo transfer

There was no significant difference between the fresh ET and FET groups with regard to the duration of ovarian stimulation. The total gonadotropin dose in fresh ET group was significantly higher than that in FET group (2274.50 ± 740.08 IU vs. 1938.81 ± 652.88 IU, *P* < 0.001). Estradiol level on hCG trigger day in fresh ET group was significantly lower compared to the FET group (3235.65 ± 1196.11 vs. 4480.45 ± 789.60 pg/ml, *P* < 0.001). Number of oocytes retrieved and available embryos in fresh ET group was significantly less than the FET group (10.49 ± 3.77 vs. 17.97 ± 5.47 and 4.00 ± 2.03 vs. 5.44 ± 2.69, *P* < 0.001). With regard to the insemination method, there was significant difference between the two groups of using IVF or ICSI (*P* < 0.001) (Table 2).

**Table 2:** Treatment characteristics in fresh ET and FET groups

Parameters	Fresh ET (*n* = 5216)	FET (*n* = 2598)	P
Ovarian stimulation (days)	10.90 ± 1.30	10.85 ± 1.28	0.180
Gonadotropin dose (IU)	2274.50 ± 740.08	1938.81 ± 652.88	< 0.001
Estradiol level on hCG trigger day (pg/ml)	3235.65 ± 1196.11	4480.45 ± 789.60	< 0.001
No. of oocytes retrieved	10.49 ± 3.77	17.97 ± 5.47	< 0.001
Insemination method			< 0.001
IVF, n (%)	4376 (83.9)	2095 (80.6)	
ICSI, n (%)	840 (16.1)	503 (19.4)	
No. of available embryos	4.00 ± 2.03	5.44 ± 2.69	< 0.001

Note: Presented as mean SD for continuous variables. *ICSI* intracytoplasmic sperm injection; *IVF* in vitro fertilization; *FET *frozen embryo transfer; *fresh ET* fresh embryo transfer

The clinical outcomes in fresh ET and FET groups were shown in Table 3. After adjusting for potential confounders including age, infertility duration, BMI, AMH, number of oocytes retrieved and available embryos, multivariate logistic regression analysis demonstrated that there was no significant difference in terms of clinical pregnancy rate (65.8 % vs. 66.9 %, *P* = 0.683), ectopic pregnancy rate (2.9 % vs. 4.2 %, *P* = 0.297) and pregnancy loss rate (9.2 % vs. 12.4 %, *P* = 0.072) between fresh ET group and FET groups. However, the implantation rate and live birth rate of fresh ET group were significantly higher in fresh ET group than that in FET group (48.6 % vs. 47.0 %, *P* < 0.001 and 57.1 % vs. 54.8 %, *P* = 0.012, respectively).

**Table 3:** Clinical outcomes in fresh ET and FET groups

Outcomes	Fresh ET (*n* = 5216)	FET (*n* = 2598)	95 % CI	P
Implantation, n (%)	5066 (48.6 %)	2433 (47.0 %)	1.103–1.315	< 0.001
Clinical Pregnancy, n (%)	3431 (65.8 %)	1737 (66.9 %)	0.918–1.139	0.683
Ectopic pregnancy, n (%)	153 (2.9 %)	109 (4.2 %)	0.601–1.168	0.297
Pregnancy loss, n (%)	316 (9.2 %)	215 (12.4 %)	0.997–1.066	0.072
Live birth, n (%)	2978 (57.1 %)	1425 (54.8 %)	1.037–1.332	0.012

Note: Presented as n (%) for categoric variables. *P* value was adjusted for age, infertility duration, BMI, AMH, No. of oocytes retrieved and No. of available embryos by multivariate logistic regression analysis. *BMI* body mass index; *AMH* Anti-Mullerian Hormone

## Discussion

The progress of embryo cryopreservation technology, particularly in vitrification, has made freeze-all strategy more acceptable. However, the freeze all strategy is still controversial due to its advantages and disadvantages assessment. There are currently no clinical data supporting the indiscriminate use of freeze-all strategy for all patients [15]. In this study, we analyzed whether patients benefited from freeze all strategy compared with fresh ET in GnRH-a long protocol. The reason why we chose the first FET cycle was that the best-quality embryos were usually transferred in the first FET which is similar in fresh ET. Simultaneously, patients whose age > 34 years, endometrium thickness < 0.7 cm, with blastocyst transfer and PGT cycles were excluded, because these confounding factors were extremely unbalanced between the two groups. The baseline characteristics of the patients including age, infertility duration, AMH level, basal FSH level, primary infertility and primary cause of infertility were still significantly different between two groups (Table 1). Especially, the younger age and higher AMH level in FET group might indicate that the function of ovarian reserve of the FET group was better than the fresh ET group. As expected, the outcomes of COS of the FET group were better, including less gonadotropin dose, more oocytes retrieved and more available embryos (Table 2). However, more available embryos would more impact the cumulative live birth rather than the LBR for the first ET.

As we mentioned, the COS protocol in all the previous studies that compared fresh ET and FET was GnRH-ant protocol. There has been no report regarding the comparison of the efficacy between fresh ET and FET with GnRH-a protocol. The reason why we chose GnRH-a protocol is because it is still the leading protocol in China. Implantation is one of the most important steps to achieve live birth, therefore it is considered as an important indicator of the efficacy of the treatment. The process of implantation involves two main components, the embryo quality and endometrial receptivity [18]. Hershko et al. have conducted a randomized trial which showed no difference in embryo quality between GnRH-a and GnRH-ant groups [19]. As for the endometrial receptivity, Hernandez et al. have reported that GnRH-ant may disrupt an auto/paracrine loop, which is essential for the mitotic programme of the endometrial epithelial cells, leading to a decline in pregnancy rates and an increase of abortion rates [20]. Rackow et al. found HOXA10 (an essential regulator of endometrial receptivity) expression was significantly decreased in endometrial stromal cells in GnRH-ant-treated cycles compared with GnRH-a-treated cycles or natural cycle [21]. Ruan et al. found GnRH agonist, may partially restore the endometrial physiological secretion and improve uterine receptivity in mice [22]. A comparative proteomic analysis demonstrated endometrial receptivity was more strongly impaired by GnRH-ant than GnRH-a treatments [23]. The results of the above studies [20–23] indicated that the endometrial receptivity of GnRH-a protocol might be better than GnRH-ant protocol in fresh ET cycles. Shi Y et al. found there were no significant differences in the rates of implantation between the frozen-embryo group and the fresh-embryo group with GnRH-ant protocol. But in this study we found that the implantation rate in the fresh ET group with GnRH-a protocol was higher than the FET group (Table 3).

As we know, FET has become increasingly common in many countries [8]. It has been hypothesized that FET may provide a more physiologic uterine environment for embryo implantation than fresh ET [24]. Furthermore, the elective freezing embryos also can reduce the risk of OHSS, which is an iatrogenic, serious, and potentially life-threatening complication in COS treatment [25, 26]. Recent studies found reduced uterine artery pulsatility index [27] and greater CRL growth [28] in FET pregnancies as compared to fresh which might decipher fresh embryo transfer newborns were smaller than FET newborns. However, it should be noted to the damage of embryo by freezing and thawing, which was associated with ice crystal formation, increased of salt concentrations and cryoprotectant agents toxicity caused by cryopreservation [29–31]. Tachataki et al. demonstrated that cryopreservation affected the normal pattern of gene expression during human pre-implantation development [32]. Therefore, when the benefits of performing FET to the uterine environment do not outweigh the damage risk of freezing and thawing to embryos, the live birth rate will be finally lower for the FET compared to fresh ET as we found in this study (Table 3). Moreover, freeze all strategy and additional frozen cycles will increase time to live birth and treatment costs for infertile patients [33]. As for obstetric complications, some studies found more frequent postpartum haemorrhage in FET than in fresh embryo transfer [34], significantly increased maternal risks of placenta accreta and pregnancy-induced hypertension [35].

There are controversial opinions regarding the ectopic pregnancy rate and pregnancy loss rate between fresh ET and FET cycles in different studies. Most researchers thought the supra physiologic hormonal levels could confer a higher ectopic pregnancy rate with fresh ET cycles [36, 37]. However, Xiao et al. found no significant difference in ectopic pregnancy rate between fresh ET and FET [38]. Chen et al. found there was higher pregnancy loss rate in the fresh ET group compared to FET group in ovulatory women [13]. However, Heather et al. found a higher first trimester pregnancy loss risk after FET compared with fresh ET among women younger than 38 years old [39]. In this cohort study, the ectopic pregnancy rate and pregnancy loss rate were higher in the FET group, but after adjusting for potential confounders, multivariate logistic regression analysis showed no significant difference between two groups.

This study has its inherent limitation as a retrospective analysis. The characteristics of patients were unbalanced. Although we adjusted the confounders and used multivariate logistic regression analysis, some potential confounders still might be ignored. In conclusion, compared to FET, fresh ET following GnRH-a long protocol tended to increase live birth rate in patients undergoing their first ART cycle. This study suggests that freeze all strategy is not for all, and it should be individualized especially for GnRH-a long protocol in clinical practice. A well-designed, multicentre, prospective RCT is still required to further support these results.

## Conclusions

Compared to FET, fresh ET following GnRH-a long protocol could lead to higher implantation rate and live birth rate in infertile patients underwent IVF. The freeze all strategy should be given some rethinking, individualized adjustment may be necessary especially with GnRH-a long protocol.

## Data Availability

The datasets generated for this study are available on request to the corresponding author.

## Abbreviations

FET: Frozen Embryo Transfer
ET: Embryo Transfer
GnRH-ant: Gonadotropin Releasing Hormone antagonist
GnRH-a: Gonadotropin Releasing Hormone agonist
IVF: In Vitro Fertilization
ICSI: Intracytoplasmic Sperm Injection
BMI: Body Mass Index
AMH: Anti-Müllerian Hormone
COS: Controlled Ovarian Stimulation
OHSS: Ovarian Hyperstimulation Syndrome
ART: Assisted Reproductive Technology
PGT: Pre-implantation Genetic Testing
RCT: Randomized Controlled Trial
FSH: Follicle Stimulating Hormone
AFC: Antral Follicle Counts
rhCG: Recombinant human Chorionic Gonadotropin

## References

[1] Fleming R, Adam AH, Barlow DH, Black WP, MacNaughton MC, Coutts JR (1982). A new systematic treatment for infertile women with abnormal hormone profiles. Br J Obstet Gynaecol.

[2] Wang R, Lin S, Wang Y, Qian W, Zhou L (2017). Comparisons of GnRH antagonist protocol versus GnRH agonist long protocol in patients with normal ovarian reserve: A systematic review and meta-analysis. PLoS One.

[3] Tomas C, Toftager M, Lossl K, Bogstad J, Praetorius L, Zedeler A (2019). Perinatal outcomes in 521 gestations after fresh and frozen cycles: a secondary outcome of a randomized controlled trial comparing GnRH antagonist versus GnRH agonist protocols. Reprod Biomed Online.

[4] Lambalk CB, Banga FR, Huirne JA, Toftager M, Pinborg A, Homburg R (2017). GnRH antagonist versus long agonist protocols in IVF: a systematic review and meta-analysis accounting for patient type. Hum Reprod Update.

[5] Roque M, Lattes K, Serra S, Sola I, Geber S, Carreras R (2013). Fresh embryo transfer versus frozen embryo transfer in in vitro fertilization cycles: a systematic review and meta-analysis. Fertil Steril.

[6] Li J, Yin M, Wang B, Lin J, Chen Q, Wang N (2020). The effect of storage time after vitrification on pregnancy and neonatal outcomes among 24 698 patients following the first embryo transfer cycles. Hum Reprod.

[7] Shapiro BS, Daneshmand ST, Garner FC, Aguirre M, Ross R (2008). Contrasting patterns in in vitro fertilization pregnancy rates among fresh autologous, fresh oocyte donor, and cryopreserved cycles with the use of day 5 or day 6 blastocysts may reflect differences in embryo-endometrium synchrony. Fertil Steril.

[8] Evans J, Hannan NJ, Edgell TA, Vollenhoven BJ, Lutjen PJ, Osianlis T (2014). Fresh versus frozen embryo transfer: backing clinical decisions with scientific and clinical evidence. Hum Reprod Update.

[9] Aflatoonian A, Mansoori Moghaddam F, Mashayekhy M, Mohamadian F (2010). Comparison of early pregnancy and neonatal outcomes after frozen and fresh embryo transfer in ART cycles. J Assist Reprod Genet.

[10] Chen ZJ, Shi Y, Sun Y, Zhang B, Liang X, Cao Y (2016). Fresh versus Frozen Embryos for Infertility in the Polycystic Ovary Syndrome. N Engl J Med.

[11] Wong KM, Mastenbroek S, Repping S (2014). Cryopreservation of human embryos and its contribution to in vitro fertilization success rates. Fertil Steril.

[12] Roque M (2015). Freeze-all policy: is it time for that?. J Assist Reprod Genet.

[13] Shi Y, Sun Y, Hao C, Zhang H, Wei D, Zhang Y (2018). Transfer of Fresh versus Frozen Embryos in Ovulatory Women. N Engl J Med.

[14] Vuong LN, Dang VQ, Ho TM, Huynh BG, Ha DT, Pham TD (2018). IVF Transfer of Fresh or Frozen Embryos in Women without Polycystic Ovaries. N Engl J Med.

[15] Roque M, Haahr T, Geber S, Esteves SC, Humaidan P (2019). Fresh versus elective frozen embryo transfer in IVF/ICSI cycles: a systematic review and meta-analysis of reproductive outcomes. Hum Reprod Update.

[16] Mathur R, Kailasam C, Jenkins J (2007). Review of the evidence base of strategies to prevent ovarian hyperstimulation syndrome. Hum Fertil (Camb).

[17] Xie X, Kong BH. Duan tao. Obstetrics and gynecology. 9th Edition. (Chinese).

[18] Diedrich K, Fauser BC, Devroey P, Griesinger G, Evian Annual Reproduction Workshop G (2007). The role of the endometrium and embryo in human implantation. Hum Reprod Update.

[19] Hershko Klement A, Berkovitz A, Wiser A, Gonen O, Amichay K, Cohen I (2015). GnRH-antagonist programming versus GnRH agonist protocol: a randomized trial. Eur J Obstet Gynecol Reprod Biol.

[20] Hernandez ER (2000). Embryo implantation and GnRH antagonists: embryo implantation: the Rubicon for GnRH antagonists. Hum Reprod.

[21] Rackow BW, Kliman HJ, Taylor HS (2008). GnRH antagonists may affect endometrial receptivity. Fertil Steril.

[22] Ruan HC, Zhu XM, Luo Q, Liu AX, Qian YL, Zhou CY (2006). Ovarian stimulation with GnRH agonist, but not GnRH antagonist, partially restores the expression of endometrial integrin beta3 and leukaemia-inhibitory factor and improves uterine receptivity in mice. Hum Reprod.

[23] Chen Q, Yu F, Li Y, Zhang AJ, Zhu XB (2019). Comparative proteomics reveal negative effects of gonadotropin-releasing hormone agonist and antagonist on human endometrium. Drug Des Devel Ther.

[24] Weinerman R, Mainigi M (2014). Why we should transfer frozen instead of fresh embryos: the translational rationale. Fertil Steril.

[25] Devroey P, Polyzos NP, Blockeel C (2011). An OHSS-Free Clinic by segmentation of IVF treatment. Hum Reprod.

[26] Zech J, Brandao A, Zech M, Lugger K, Neururer S, Ulmer H (2018). Elective frozen-thawed embryo transfer (FET) in women at risk for ovarian hyperstimulation syndrome. Reprod Biol.

[27] Cavoretto PI, Farina A, Gaeta G, Sigismondi C, Spinillo S, Casiero D (2020). Uterine artery Doppler in singleton pregnancies conceived after in-vitro fertilization or intracytoplasmic sperm injection with fresh vs frozen blastocyst transfer: longitudinal cohort study. Ultrasound Obstet Gynecol.

[28] Cavoretto PI, Farina A, Girardelli S, Gaeta G, Spinillo S, Morano D, et al Greater fetal crown-rump length growth with the use of in vitro fertilization or intracytoplasmic sperm injection conceptions after thawed versus fresh blastocyst transfers: secondary analysis of a prospective cohort study. *Fertil Steril* (2021); S0015-0282(20)32703-5. DOI: 10.1016/j.fertnstert.2020.11.035. 10.1016/j.fertnstert.2020.11.035 33500139

[29] Mansoori GA (1975). Kinetics of water loss from cells at subzero centigrade temperatures. Cryobiology.

[30] Kleinhans FW, Mazur P (2007). Comparison of actual vs. synthesized ternary phase diagrams for solutes of cryobiological interest. Cryobiology.

[31] Pinborg A, Henningsen AA, Loft A, Malchau SS, Forman J, Andersen AN (2014). Large baby syndrome in singletons born after frozen embryo transfer (FET): is it due to maternal factors or the cryotechnique?. Hum Reprod.

[32] Tachataki M, Winston RM, Taylor DM (2003). Quantitative RT-PCR reveals tuberous sclerosis gene, TSC2, mRNA degradation following cryopreservation in the human preimplantation embryo. Mol Hum Reprod.

[33] Polyzos NP, Drakopoulos P, Parra J, Pellicer A, Santos-Ribeiro S, Tournaye H (2018). Cumulative live birth rates according to the number of oocytes retrieved after the first ovarian stimulation for in vitro fertilization/intracytoplasmic sperm injection: a multicenter multinational analysis including approximately 15,000 women. Fertil Steril.

[34] Choux C, Ginod P, Barberet J, Rousseau T, Bruno C, Sagot P, rt al. Placental volume and other first-trimester outcomes: are there differences between fresh embryo transfer, frozen-thawed embryo transfer and natural conception? *Reprod Biomed Online* (2019); 38:538–548. doi: 10.1016/j.rbmo.2018.12.023. 10.1016/j.rbmo.2018.12.023 30850320

[35] Ishihara O, Araki R, Kuwahara A, Itakura A, Saito H, Adamson GD (2014). Impact of frozen-thawed single-blastocyst transfer on maternal and neonatal outcome: an analysis of 277,042 single-embryo transfer cycles from 2008 to 2010 in Japan. Fertil Steril.

[36] Zhang X, Ma C, Wu Z, Tao L, Li R, Liu P (2018). Frozen-Thawed Embryo Transfer Cycles Have a Lower Incidence of Ectopic Pregnancy Compared With Fresh Embryo Transfer Cycles. Reprod Sci.

[37] Xing W, Ou J, Cai L (2018). Thawed embryo transfer and ectopic pregnancy: a meta-analysis. Arch Gynecol Obstet.

[38] Xiao S, Mo M, Hu X, Zhang H, Xu S, Wang Z (2018). Study on the incidence and influences on heterotopic pregnancy from embryo transfer of fresh cycles and frozen-thawed cycles. J Assist Reprod Genet.

[39] Hipp H, Crawford S, Kawwass JF, Chang J, Kissin DM, Jamieson DJ (2016). First trimester pregnancy loss after fresh and frozen in vitro fertilization cycles. Fertil Steril.

